# Photoconverted cells allow rapid assessment of vaccine adjuvant potency in mice

**DOI:** 10.1016/j.isci.2025.112774

**Published:** 2025-06-05

**Authors:** Yiwei Zhong, Mingyue Chen, Hongzhe Lin, Cheng Zu, Yue He, Bin Wang

**Affiliations:** 1 MOE/NHC/CAMS Key Laboratory of Medical Molecular Virology, Shanghai Institute of Infectious Disease and Biosecurity, Shanghai Frontiers Science Center of Pathogenic Microorganisms and Infection, School of Basic Medical Sciences, Shanghai Medical College, Fudan University, Shanghai 200032, China; 2 Shanghai Institute of Infectious Disease and Biosecurity, Fudan University, Shanghai 200032, China; 3 Advaccine Biopharmaceuticals (Suzhou) Co. Ltd, Suzhou 215000, China

**Keywords:** Immunology, Methodology in biological sciences

## Abstract

Identifying vaccine adjuvants that optimally enhance CD8^+^ T cell responses remain a challenge, often requiring time-consuming and resource-intensive methods. Here, we introduce a photoconversion-based approach using KikGR mice to track migratory dendritic cells (Mig DCs) within 48 h post-vaccination. This method enables real-time visualization of skin-derived Mig DCs migration to draining lymph nodes (dLNs), providing an early and reliable predictor of CD8^+^ T cell priming and anti-tumor efficacy. Unlike traditional techniques that demand extensive experimentation, this system allows for faster, cost-effective screening of adjuvants. We further demonstrate that blocking CCR7 significantly reduces Mig DCs migration, impairing CD8^+^ T cell responses. Our approach revolutionizes adjuvant evaluation, enabling swift and accurate immune assessments, particularly in therapeutic vaccine development.

## Highlights

• Migratory DCs serve as predictor for rapid assessment of adjuvants’ potency
• The KikGR mice offer real-time tracking of migratory DCs to dLNs
• The quantity of KikGR-red cells correlates with CD8^+^ T cell responses

## Graphical abstract

**Figure:**
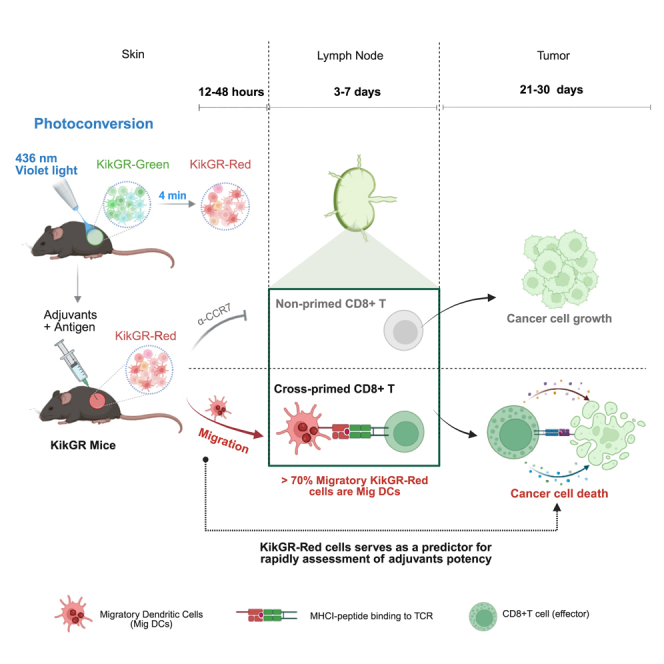

## Introduction

Vaccine adjuvants are critical components for enhancing the adaptive immune response of vaccination, particularly for diseases such as SARS-CoV-2, hepatitis B, and influenza.^1^^,^^2^^,^^3^ Traditional adjuvants, such as aluminum salt (Alum) and MF59, are effective in boosting humoral immune responses.^4^^,^^5^ However, recent advancements have focused on the adjuvants, particularly those that target Toll-like receptors (TLRs) such as CpG1018 (TLR9 agonist) and R848 (TLR7/8 agonist) or combinations.^6^^,^^7^^,^^8^^,^^9^ Additionally, TLR3 and TLR4 agonists, including poly I:C and monophosphoryl lipid A (MPLA), respectively, have demonstrated enhanced immune responses.^10^^,^^11^ The emergence of Stimulator of Interferon Genes (STING) pathway agonists has offered significant antiviral and antitumor responses, marking a substantial advance in adjuvant technology.^12^

While most prophylactic vaccines excel at boosting humoral immunity, their ability to stimulate robust CD8^+^ T cell responses remains a challenge. CD8^+^ T cells are pivotal for generating cytotoxic immune responses, particularly in cancer and therapeutic vaccine settings.^13^^,^^14^ Although adjuvants like CpG and R848 can enhance CD8^+^ T cell responses, optimizing their magnitude, quality, and durability for various applications is crucial. There is significant interest in developing the adjuvants that can reliably elicit strong and durable CD8^+^ T cell responses, which are essential for protection against various pathogens and for effective anti-tumor immunity. Current methods for screening adjuvants often require extensive experimentation, prolonged component selections, dose optimization, and lengthy efficacy studies.^15^^,^^16^ Thus, a faster, reliable method to predict the ability of adjuvants to enhance CD8^+^ T cell responses would significantly accelerate vaccine development.

Dendritic cells (DCs) are professional antigen-presenting cells in the immune system, playing a critical role in initiating adaptive T cell responses. Migration of DCs to draining lymph nodes (dLNs) is essential for priming T responses.^17^ Following subcutaneous immunization, two distinct DC populations contribute to antigen delivery in dLNs. Resident lymph node DCs (Res DCs) enter LNs from the bloodstream and acquire antigen via either lymphatic drainage or from other cells, while skin-derived migratory DCs (Mig DCs) capture antigen directly in peripheral tissues and migrate to dLNs.^18^ Notably, Mig DCs, particularly migratory type 1 conventional DC subset (Mig cDC1), aggregate more rapidly than the Res DCs in dLNs within the first 2 days after immunization and are critical for cross-priming naive CD8^+^ T cells.^19^^,^^20^^,^^21^^,^^22^ However, distinguishing Mig DCs from Res DCs during adjuvant-induced inflammation remains a challenge due to overlapping phenotypes and functional plasticity.^23^^,^^24^

To track Mig DCs in dLNs, we employed the photoconversion KikGR mouse model. In this system, *KikGR-red cells predominantly represent Mig DCs, though they may also include Langerhans cells as previously documented.*^25^ We hypothesized that the number of the photoconverted KikGR-red cells migrating into the dLNs directly correlated with the magnitude of the CD8^+^ T cell response. This approach enables real-time tracking of Mig DCs during immune activation, providing insights into their role in shaping adaptive immunity.

The KikGR mouse is a transgenic animal with a fluorescent protein engineered for green-to-red photoconvertibility via ultraviolet or violet light irradiation.^26^ The KikGR protein contains a His62-Tyr63-Gly64 tripeptide sequence, which forms a green chromophore. Photoconversion induces formal β-elimination and extension of a π-conjugated system, resulting in a stable red chromophore.^27^ This mechanism allows precise labeling cells *in vivo*, facilitating their visualization during migration.

In this study, we present a method using KikGR photoconvertible mice to track and quantify the migration of skin-derived Mig DCs in response to vaccination. The KikGR mouse model allows for real-time tracking of these cells by converting their fluorescence from green to red upon exposure to violet light.^28^^,^^29^^,^^30^ This photoconversion technique provides a unique opportunity to monitor the dynamic migration and turnover of Mig DCs, facilitating an efficient and quantitative evaluation of adjuvant-induced immune responses. Our results demonstrate that the amplitude of KikGR-red cells in dLNs correlates strongly with CD8^+^ T cell priming and anti-tumor efficacy. Importantly, we show that blocking CCR7, a key chemokine receptor guiding DC migration, significantly reduces KikGR-red cells, impairing CD8^+^ T cell responses. This study highlights the potential of KikGR-red cells as early predictors of CD8^+^ T cell activation and offers a rapid and cost-effective approach to screen adjuvants.

By employing this photoconversion system, we offer an accelerated method to evaluate adjuvants targeting CD8^+^ T cell responses within 48 h post-immunization. This approach is particularly advantageous in therapeutic vaccine development, where time is critical for optimizing immune response efficacy.

## Results

### Photoconversion tracking skin cells in KikGR mice

**Figure 1:**
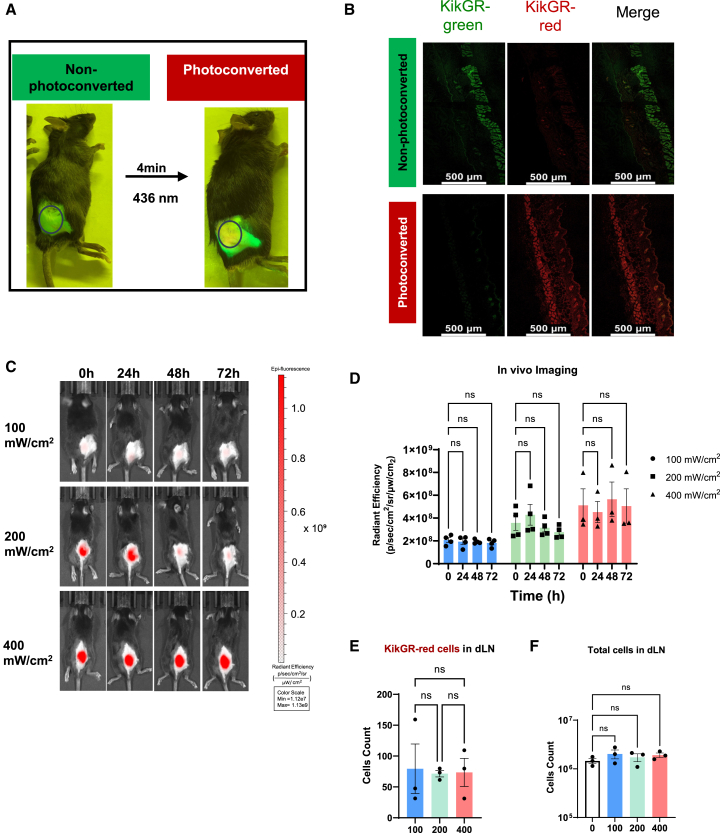
Photoconversion of skin cells in KikGR mice (A) The hair-clipped skin of female KikGR mice was exposed to violet light (436 nm, 200 mW/cm^2^ intensity) for 4 min to induce photoconversion. The KikGR mouse before and after photoconversion was illuminated using the hand-held lamp and observed through a green filter. (B) The photoconverted and non-photoconverted skin was harvested from mice 24 h after illumination at an intensity of 200 mW/cm^2^ for 4 min. Confocal microscopy shows photoconversion of KikGR-green and KikGR-red fluorescence by Leica Stellaris 8 laser scanning confocal microscope using appropriate lase and filter sets. Scale bars represent 500 μm. (C and D) The hair-clipped skin of KikGR mice was photoconverted by violet light at intensities of 100, 200, and 400 mW/cm^2^, respectively, and KikGR-red fluorescence was measured via living image at 0, 24, 48, and 72 h post-photoconversion (C). Radiant efficiency was analyzed. Data shown are mean ± SEM from two independent experiments (100 mW/cm^2^, *n* = 4; 200 mW/cm^2^, *n* = 4; 400 mW/cm^2^, *n* = 3). ns, not significant by two-way ANOVA (D). (E and F) The number of KikGR-red cells in dLN (E) and total dLN cells (F) were analyzed at 3 h post-photoconversion at intensities of 100, 200, or 400 mW/cm^2^, with no photoconversion serving as a negative control. Data shown are mean ± SEM from two independent experiments (*n* = 3 mice/group). ns, not significant by one-way ANOVA (E and F).

In our experiments, the hair-clipped skin of KikGR mice was exposed to violet light at 436 nm with an intensity of 200 mW/cm^2^ for 4 min to induce photoconversion. Figure 1A shows a KikGR mouse before and after photoconversion. The mouse was illuminated by a hand-held lamp and observed through a green filter, allowing us directly visualize the KikGR-red signal on the exposed skin region. Confocal microscopy confirmed the complete transformation of KikGR-green to KikGR-red within the treated skin area at the 24-h post-photoconversion (Figure 1B). Notably, there was no detectable KikGR-green in the exposed skin section compared to the non-exposed region (Figure 1B), demonstrating the efficiency of this photoconversion method for labeling skin cells in experimental settings.

Upon adjuvant stimulation, the accumulation of Mig DCs in the dLNs is typically observed for 1–2 days. A challenge arises during this period as the KikGR-red signal diminishes due to the cells’ ongoing expression of the KikGR-green gene.^31^^,^^32^ To address this issue and ensure consistent differentiation between KikGR-green and KikGR-red cells, we increased the light intensity for skin photoconversion near the dLN from 100 mW/cm^2^ to 400 mW/cm^2^. On days 1, 2, and 3 after photoconversion, KikGR-green fluorescence was acquired using an *in vivo* imaging system at excitation/emission (EX/EM) wavelength 465 nm/520 nm, and KikGR-red fluorescence was acquired at EX/EM wavelength 535 nm/600 nm. The results revealed that a light intensity of 400 mW/cm^2^ elicited a stronger KikGR-red signal compared to lower intensities of 100 mW/cm^2^ and 200 mW/cm^2^. Notably, the KikGR-red signal was effectively maintained for an extended period of 72 h (3 days) at 400 mW/cm^2^ without any observable decline (Figures 1C and 1D). We analyzed CD45^+^ cells in the photoconverted skin region 24 h post-photoconversion using flow cytometry (FACS). As shown in the Figure S1, most CD45^+^ cells appeared in the KikGR-green and KikGR-red double-positive quadrant. As expected, we observed an enhanced KikGR-red signal in CD45^+^ cells with elevated light intensity. Importantly, this adjustment did not lead to an excessive influx of KikGR-red cells into the dLNs, nor did it cause dLNs swelling compared to non-exposed skin dLNs (Figure 1E and 1F). These findings indicate that using a light intensity of 400 mW/cm^2^ is a safe and effective method for visualizing the migration of Mig DCs into the dLN for at least 3 days, without adversely affecting cell behavior or lymph node physiology.

### Translocation of KikGR-red cells correlated to the migratory DCs

The migration of antigen-sampled DCs to dLNs is crucial for triggering immune responses. This study utilized KikGR-red fluorescence to monitor the dynamic migration of these cells in response to inflammation induced by an antigen coupled with various adjuvants. The experimental procedure involved photoconverting the skin of KikGR mice using 436 nm light at 400 mW/cm^2^ for 4 min, followed by subcutaneous immunization at the converted sites with ovalbumin (OVA) protein combined with different adjuvants: TLR agonists CR108 (TLR9 and TLR7/8), Aluminum (Alum), and MF59.

**Figure 2:**
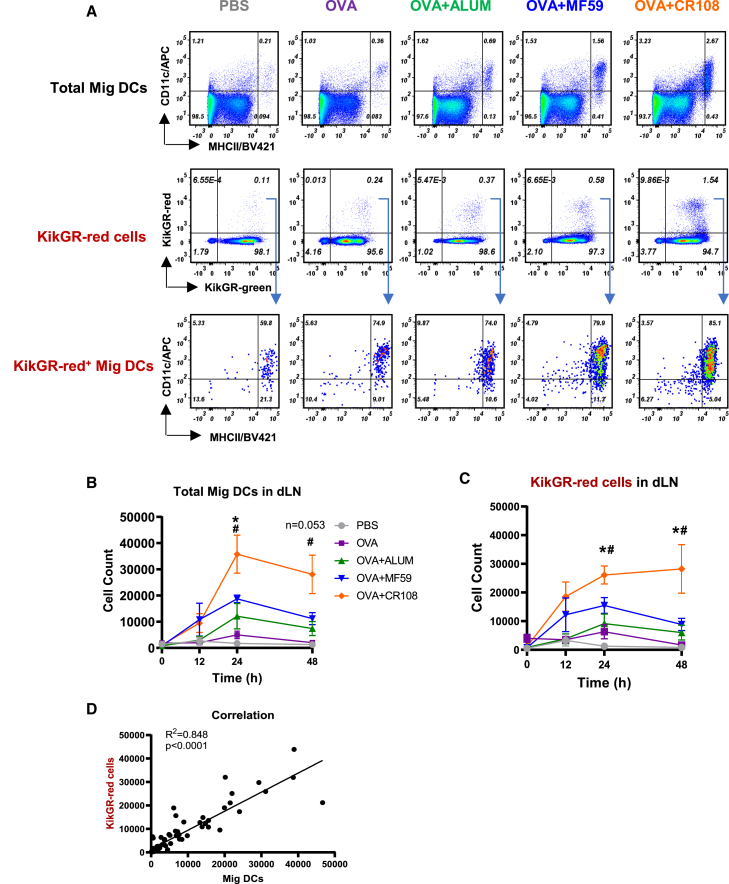
FACS analysis of dynamic patterns of KikGR-red cells and Mig DCs activated by various adjuvants (A) KikGR mice with hair-clipped skin were photoconverted using 436 nm violet laser light at an intensity of 400 mW/cm^2^ for 4 min. Three hours later, CR108 + OVA, MF59 + OVA, Alum + OVA, OVA alone, or PBS (as a control) were administrated to the photoconverted skin site. Total MHC-II^hi^CD11c^med^ cells (Mig DCs), KikGR-red cells and MHCII^hi^CD11c^med^ cells in KikGR-red cells within dLNs were determined by flow cytometry 24 h after administration. (B–D) The dynamic number of total Mig DCs in each whole dLN was analyzed by flow cytometry at 0, 12, 24, and 48 h post-administration by anti-MHCII and anti-CD11c antibodies. (C) Using the same animals with (B), the dynamic, precise number of KikGR-red cells in each whole dLN were also measured at 0, 12, 24, and 48 h post-administration. Data are mean ± SEM from two independent experiments (At 0, 12, 24, and 48 h, PBS: *n* = 4, 4, 3, 3; OVA: *n* = 4, 3, 4, 3; Alum + OVA: *n* = 4, 4, 4, 4; MF59 + OVA: *n* = 4, 4, 4, 4; CR108 + OVA *n* = 4, 4, 3, 3). Statistical significances: #*p* < 0.05 (CR108 + OVA vs. Alum + OVA), ∗*p* < 0.05 and ns (not significant) (CR108 + OVA vs. MF59 + OVA) by Student’s *t* test. (D) Correlation between KikGR-red cell counts and MHC-II^hi^CD11c^med^ Mig DCs in dLNs was analyzed.

Analysis at 24 h post-immunization revealed that a significant proportion (approximately 70%–80%) of KikGR-red cells within the dLN during adjuvant-triggered inflammation belonged to the CD11c^med^MHCII^hi^ Mig DCs subset. Conversely, administration of PBS as a control resulted in a minimal presence of Mig DCs among KikGR-red cells in the dLN (Figure 2A), *whereas* no KikGR-red signal was observed in unconverted KikGR mice in dLNs by *FACS analysis* (Figure S2). Different adjuvants exhibited distinct effects on Mig DCs migration to dLNs. CR108+OVA-activated Mig DCs showed a substantial increase in numbers starting as early as 12 h post-immunization, peaking at 24–48 h with an estimated influx of around 30,000 cells. In contrast, MF59+OVA-activated Mig DCs peaked at 24 h with an estimated influx of 15,000 cells, followed by a rapid decline by 48 h. Similarly, Alum + OVA induced far fewer cell influxes (Figure 2B).

A strong correlation was observed between Mig DCs counts and KikGR-red cells following the administration of diverse adjuvants with OVA or OVA alone (Figure 2C). This correction was analyzed by Spearman’s correlation coefficient between KikGR-red cells and Mig DCs (Figure 2D), underscoring the utility of KikGR mice as a valuable tool for studying the dynamic behavior of Mig DCs following vaccination and adjuvant exposure.

### Effects of adjuvants on KikGR-red cell cross-presentation to CD8^+^ T cells

External protein antigens are typically processed via the MHC-II pathway but can be cross-presented via MHC-I after being engulfed in DCs. Cross-presentation is crucial for enabling immune cells to sample and present exogenous antigens on MHC- I molecules, thereby initiating CD8^+^ T cell responses against various pathogens and tumors. We investigated the potential of adjuvant-activated KikGR-red cells to cross-present protein antigens, leveraging the correlation between KikGR-red cells accumulation and CD8^+^ T response.

**Figure 3:**
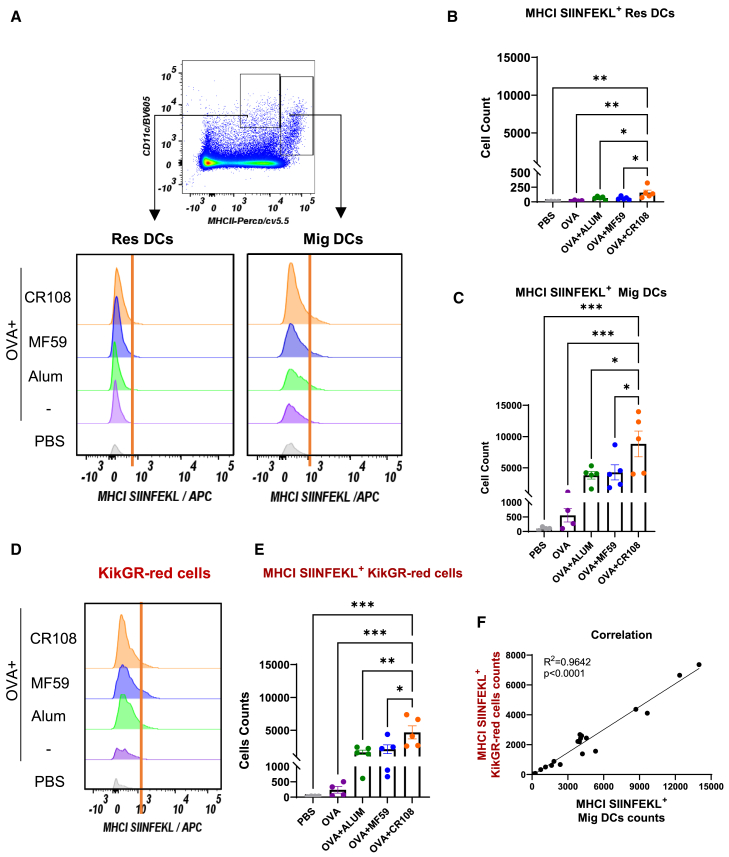
Antigen cross-presentation by KikGR-red cells activated by adjuvanted OVA (A) Experimental setup: The hair-clipped skin of KikGR mice was exposed to 436 nm violet light at an intensity of 400 mW/cm^2^ for 4 min. Three hours post-exposure, the photoconverted skin was treated with CR108 + OVA, MF59 + OVA, Alum + OVA, OVA alone, or PBS as a vehicle control. The populations of MHC-II^med^CD11c^hi^ Res DCs and MHC-II^hi^CD11c^med^ Mig DCs in dLNs were analyzed by flow cytometry 48 h post-treatment (top panel). The expression level of the MHC-I OVA_257–264_ (SIINFEKL) complex was evaluated on MHC-II^med^CD11c^hi^ Res DCs (bottom left) and MHC-II^hi^CD11c^med^ Mig DCs (bottom right) using anti-MHC-I SIINFEKL antibodies. (B–E) Quantification of antigen presentation: number of MHC-I SIINFEKL^+^ Res DCs (B) and number of MHC-I SIINFEKL^+^ Mig DCs in dLNs were quantified (C). Expression of MHC-I OVA_257–264_ (SIINFEKL) complex on KikGR-red cells in dLNs was determined to assess antigen cross presentation by FACS 48 h after administration (D). Number of MHC-I SIINFEKL^+^ KikGR-red cells was measured (E). Data shown are representative of two independent experiments with 4–5 mice in each experiment (PBS, *n* = 4; OVA, *n* = 4; Alum + OVA, *n* = 5; MF59 + OVA, *n* = 5; CR108 + OVA, *n* = 5) (mean ± SEM). ∗∗∗∗*p* < 0.0001, ∗∗∗*p* < 0.001, ∗∗*p* < 0.01, ∗*p* < 0.05, ns (not significant) by one-way ANOVA (B, C, E). (F) The correlation between MHC-I SIINFEKL^+^ KikGR-red cells and MHC-I SIINFEKL^+^ Mig DC was analyzed.

To address this, we employed an anti-OVA_257–264_ (SIINFEKL) antibody bound to H-2Kb to specifically target MHC-I molecules presenting the CD8^+^ T cell epitope OVA_257–264_. This approach enabled detection of cross-presented antigens on KikGR-red cells. Our results (Figure S3) showed that KikGR-red cells exhibited significantly higher cross-presentation levels (5%–15%) compared to KikGR-green (non-photoconverted) cells (<0.5%) post immunization. Simultaneously, our analysis revealed that Mig DCs were dominant population engaged in cross-presentation. Activation with CR108, MF59 or Alum adjuvants consistently increased the prevalence of cross-presenting Mig DCs compared to Res DCs, as illustrated in Figures 3A–3C. This suggests that KikGR-red cells mirrored as Mig DCs are the primary mediators of CD8^+^ T cell priming in the early time point.

Furthermore, CR108-activated KikGR-red cells exhibited significantly higher cross-presenting cell numbers than MF59- or Alum-activated counterparts (Figures 3D and 3E). This trend correlated proportionally Mig DCs abundance among KikGR-red cells (Figure 3F). Together, these findings highlight the crucial role of KikGR-red cells and Mig DCs in the process of antigen cross-presentation, underscoring their importance in adjuvant-driven immune response.

### Effects of adjuvants on KikGR-red cell cross-priming to CD8^+^ T cells *in vitro*

**Figure 4:**
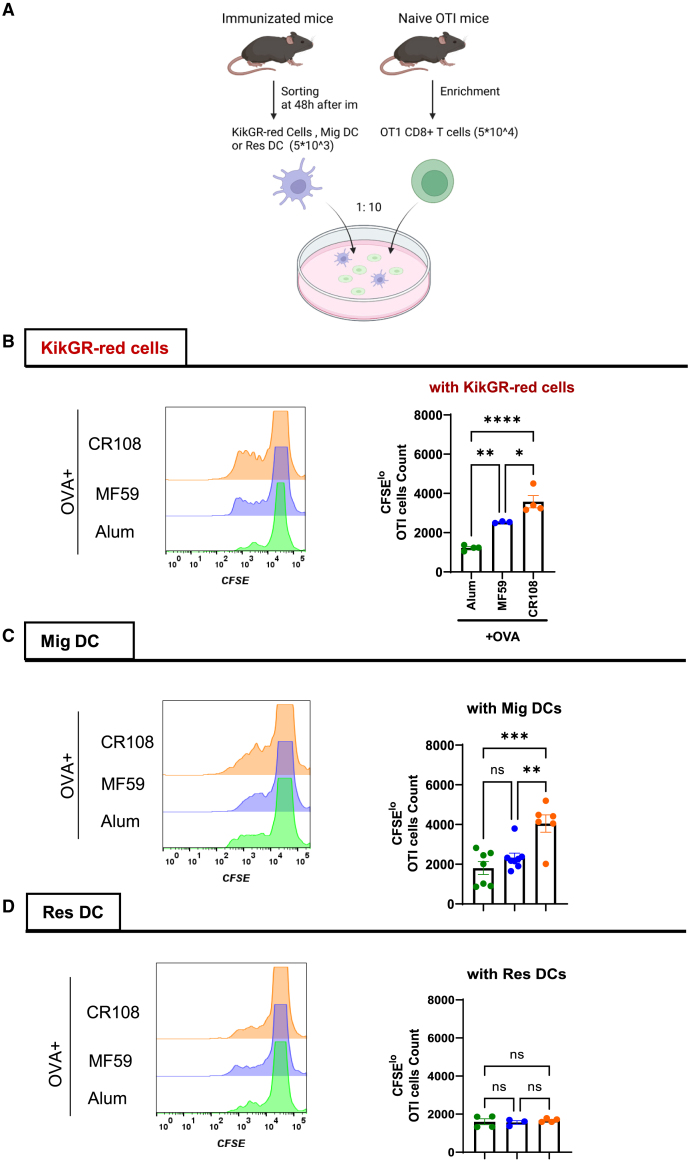
Activation of CD8^+^ T cells primed by adjuvanted OVA via activated KikGR-red cells (A) Experimental design: the hair-clipped skin of KikGR mice was exposed to 436 nm violet light at an intensity of 400 mW/cm^2^ for 4 min. Three hours later, the photoconverted skin site was treated with CR108 + OVA, MF59 + OVA, Alum + OVA, OVA alone, or PBS as a vehicle control. Forty-eight hours post-administration, KikGR-red cells, MHCII^hi^CD11c^med^ Mig DCs, and MHCII^med^CD11c^hi^Res DCs were isolated from the dLNs using FACS sorting. Naive OT-I CD8^+^ T cells at 5 × 10^4^ per well isolated from OT-I mice were used to co-culture with either 5 × 10^3^ of KikGR-red, MHC-II^hi^CD11c^med^ Mig DCs, or MHC-II^med^CD11c^hi^ Res DCs per well, respectively. (B–D) CD8^+^ T cell activation: three days post-co-culture, the number of CFSE^lo^ CD45.1^+^CD3^+^CD8^+^TCRVα2^+^ T cells co-cultured with KikGR-red cells (B), MHC-II^hi^CD11c^med^ Mig DCs (C), or MHC-II^med^CD11c^hi^ Res DCs (D) was measured by FACS. Data shown in (B) and (D) are representative of two independent experiments with 3–4 mice in each experiment (Alum + OVA, *n* = 4; MF59 + OVA, *n* = 3; CR108 + OVA, *n* = 4) (mean ± SEM). Data shown in (C) are representative of two independent experiments with 6–8 mice in each experiment (Alum + OVA, *n* = 7; MF59 + OVA, *n* = 8; CR108 + OVA, *n* = 6) (mean ± SEM). ∗∗∗∗*p* < 0.0001, ∗∗∗*p* < 0.001, ∗∗*p* < 0.01, ∗*p* < 0.05, ns (not significant) by one-way ANOVA (B, C, D).

To evaluate whether adjuvants enhance cross-presentation of protein antigens, KikGR mice were subcutaneously immunized with CR108 + OVA, MF59 + OVA, Alum + OVA, or OVA alone at photoconverted skin sites (Figure 4A). Forty-eight hours post-immunization, dLNs were harvested, and 5 × 10^3^ KikGR-red cells were sorted by FACS using KikGR-red fluorescence as the sole marker. Equivalent numbers of Mig DCs and Res DCs were isolated from dLNs using CD11c and MHC-II markers, respectively (Figure 4A).

OT-I OVA_257–264_-specific CD8^+^ T cells (5 × 10^4^ cells per well) were co-cultured with KikGR-red cells, Mig DCs, or Res DCs (Figure 4A). Flow cytometry analysis revealed that KikGR-red cells activated with CR108 + OVA induced the highest proliferation of OT-I CD8^+^ T cells, as indicated by the greatest frequency of CFSE^low^ cells (Figure 4B). This was followed by MF59 + OVA and Alum + OVA showed less proliferation. These results demonstrate that adjuvant potency directly enhances cross-presentation by KikGR-red cells, resembling the behavior of Mig DCs rather than Res DCs (Figure 4C). Consistently, Res DCs isolated from dLNs showed no significant differences in cross-presentation across adjuvant groups (Figure 4D). Overall, these findings emphasize the influence of adjuvant intensity on the cross-presentation abilities of KikGR-red cells and highlight the distinct behaviors of Mig DCs and Res DCs in the early stages of the immune response.

### Effects of adjuvants on elicitation of CD8^+^ T cell priming *in vivo*

**Figure 5:**
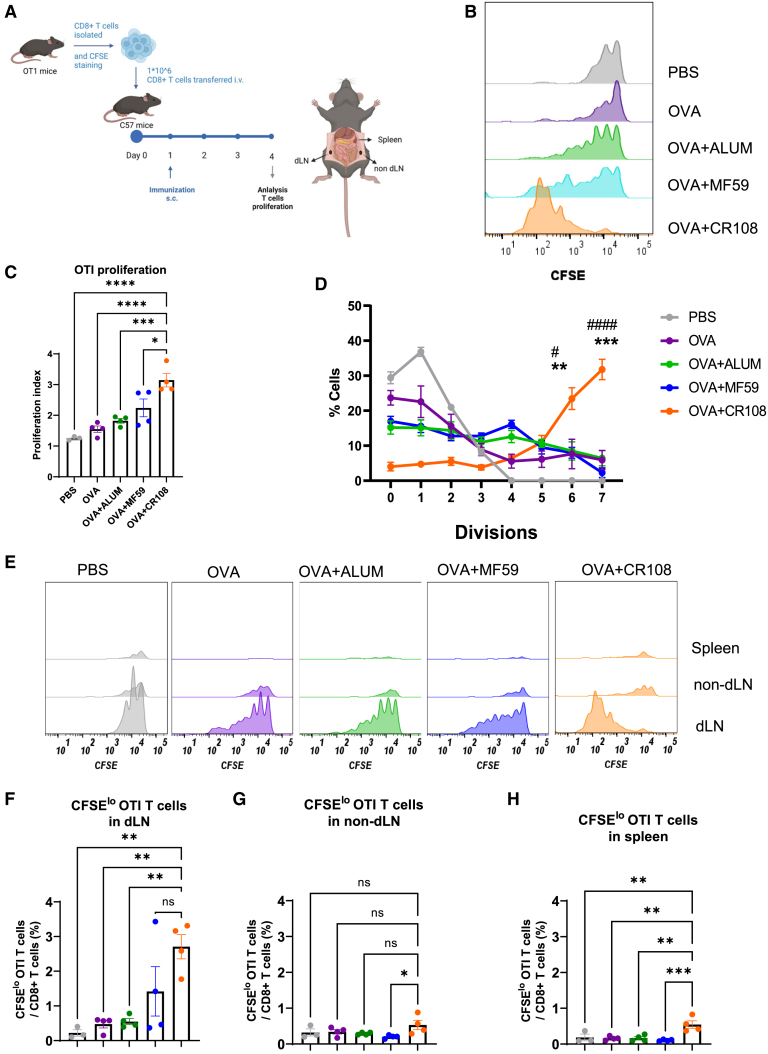
Activation of CD8^+^ T Cells following immunization with various adjuvanted OVA (A) Experimental design: CFSE-labeled OT-I CD3^+^CD8^+^ T cells (1 × 10^6^) were isolated from donor animals and adoptively transferred intravenously into naive CD45.2 C57BL/six mice on day 0. The recipient mice were then immunized subcutaneously (s.c.) on day 1 with one of the following: CR108 + OVA, MF59 + OVA, Alum + OVA, OVA alone, or PBS as a vehicle control. Three days post-immunization, CD45.1^+^TCRVα2^+^CD8^+^ T cell proliferation was assessed within draining lymph nodes (dLNs), non-draining lymph nodes (non-dLNs), and spleen via CFSE-dilution using flow cytometry. (B, C, and D) CFSE-dilution analysis in dLNs: histogram overlays depict CFSE-dilution of CD45.1^+^TCRVα2^+^CD8^+^ T cells in dLNs for each immunization group, illustrating the extent of T cell proliferation (B). (C) Proliferation index calculation: The T cell proliferation index was determined to quantify the overall proliferation response across different immunizations. (D) Proliferation in CFSE-division peaks: the percentage of CD45.1^+^TCRVα2^+^CD8^+^ T cells in each CFSE-division peak within dLNs was analyzed. Statistical significances: ^#^*p* < 0.05, and ^####^*p* < 0.0001 (CR108 + OVA versus Alum + OVA), ∗∗*p* < 0.01 and ∗∗∗*p* < 0.001 (CR108 + OVA versus MF59 + OVA) by Student’s *t* test, respectively. (E) Proliferative profiles in lymphoid organs: histogram overlays showing CFSE-dilution profiles of CD45.1^+^TCRVα2^+^CD8^+^ T cells in dLNs, non-dLNs, and spleens for all experimental groups, highlighting the differential T cell proliferation across these tissues. (F, G, and H) CD8 T cell proliferation in lymphoid tissues: the percentages of CD45.1^+^ TCRVα2^+^CD8^+^ T cells in dLNs (F), non-dLNs (G), and spleens (H) were quantified. Data shown are representative of two independent experiments with 3–4 mice in each experiment (PBS, *n* = 3; OVA, *n* = 4; Alum + OVA, *n* = 4; MF59 + OVA, *n* = 4; CR108 + OVA, *n* = 4) (mean ± SEM). ∗∗∗∗*p* < 0.0001, ∗∗∗*p* < 0.001, ∗∗*p* < 0.01, ∗*p* < 0.05, ns (not significant) by one-way ANOVA (C, F, G, H).

To test the hypothesis that increased migration of KikGR-red cells into dLNs enhances CD8^+^ T cell priming, we used syngeneic OT-I mice as a par model for adoptive transfer experiments. dLNs were defined as inguinal lymph nodes on the same side as the injection site, while non-dLNs were inguinal lymph nodes located on the opposite side.^33^ CFSE-labeled OT-I CD8^+^ T cells (1 × 10^6^ cells per mouse), specific for the OVA_257-264_ epitope, were adoptively transferred into naive C57BL/6 mice. Within 24 h, mice were vaccinated subcutaneously with CR108 + OVA, MF59 + OVA, Alum + OVA, OVA alone, or PBS as a control. Seventy-two hours post-vaccination, dLNs were harvested to assess the proliferation of OVA-specific OT-I CD8^+^ T cells (Figure 5A).

Our analysis revealed that mice immunized with OVA+CR108 exhibited the highest proliferation of CFSE-labeled OT-I CD8^+^ T cells within the dLN compared to those immunized with OVA + MF59, OVA + Alum, or OVA alone (Figures 5B and 5C). Specifically, the proliferation induced by OVA+CR108 was the most pronounced, followed by OVA + MF59, OVA + Alum, and OVA alone. Further detailed analysis indicated that in mice treated with OVA + CR108, CFSE-labeled CD8^+^ T cells underwent 6 or 7 cell divisions, significantly exceeding the 1–3 divisions observed in the other groups (Figure 5D).

In contrast, the spleen and non-dLNs showed a diminished percentage of OT-I CD8^+^ T cells and minimal proliferation across all immunization regimens (Figures 5E–5H). This distribution pattern likely results from the preferential migration of antigen-presenting cells from the injection site to the dLN, facilitating local priming of CD8^+^ T cells at an earlier stage.

### Efficacy of adjuvants on anti-tumor

To evaluate the anti-tumor efficacy of adjuvants in promoting CD8^+^ T cell-mediated tumor elimination, we utilized an OVA-expressing E.G7 tumor model in C57BL/6 mice. Mice were injected subcutaneously with 1 × 10^6^ E.G7-OVA tumor cells. Once tumors exceeded a volume of 20 mm^3^, they were treated with CR108+OVA, Alum+OVA, MF59+OVA, or OVA alone on days 1, 4, and 7 (Figure 3A).

**Figure 6:**
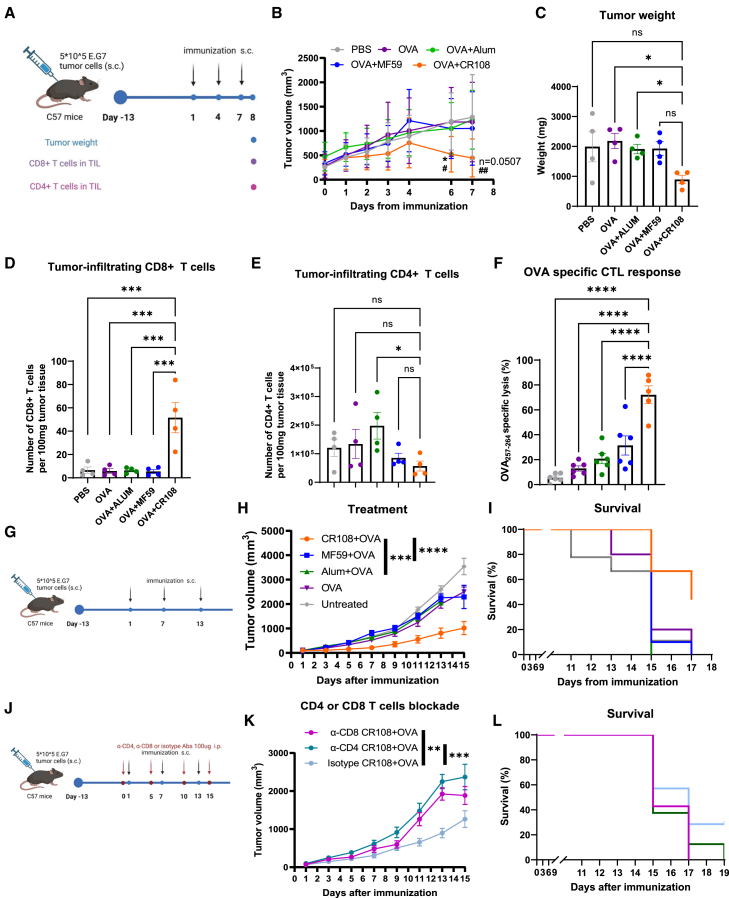
Induction of anti-tumor immune responses by various adjuvanted OVA (A–E) Schematic of representation of the experimental design. Naive female C57BL/six mice were inoculated s.c. with E.G7-OVA cells (5∗10^5^) in the right flank. Once tumor reached to approximately 20 mm^3^, CR108, MF59, Alum plus OVA, and OVA alone were administrated near the tumor sites, respectively, with PBS as a vehicle control from days 1, 4, and 7 (A). Average growth curves shown are mean ± SEM from two independent experiments (PBS, *n* = 8; OVA, *n* = 8; Alum + OVA, *n* = 8; MF59 + OVA, *n* = 8; CR108 + OVA, *n* = 9) (B). Statistical significances: # (CR108 + OVA vs. Alum + OVA, *p* < 0.05), ##(CR108 + OVA vs. Alum + OVA, *p* < 0.01) ∗(CR108 + OVA vs. MF59 + OVA, *p* < 0.05), and ns (CR108 + OVA vs. MF59 + OVA, not significant) by Student’s *t* test (C). The tumor weights were determined for each group. Quantification of tumor-infiltrating CD45^+^CD3^+^CD8^+^ T cells (D), and CD45^+^CD3^+^CD4^+^ T cells were determined by FACS at the end of the experiment (E) (Figure 3A). Data shown are representative of two independent experiments with four mice in each experiment (mean ± SEM). ∗∗∗∗*p* < 0.0001, ∗∗∗*p* < 0.001, ∗∗*p* < 0.01, ∗*p* < 0.05, ns (not significant) by one-way ANOVA (C, D, E). (F) *In vivo* CTL response measurement: C57BL/six mice were subcutaneously immunized with CR108 + OVA, MF59 + OVA, Alum + OVA, OVA alone, or PBS on days 0 and 14. On day 21 post-first immunization. Cells pulsed with OVA_257–264_ peptides were stained with CFSE^high^, while unpulsed cells were stained with CFSE^low^. CFSE^high^, and CFSE^low^ cells were mixed in 1:1 and then intravenously transferred into the immunized mice. Twenty hours later, splenocytes were analyzed by flow cytometry to assess OVA-specific lysis. Data shown are representative of two independent experiments with 5–6 mice in each experiment (PBS, *n* = 6; OVA, *n* = 6; Alum + OVA, *n* = 6; MF59 + OVA, *n* = 6; CR108 + OVA, *n* = 5) (mean ± SEM). ∗∗∗∗*p* < 0.0001, ∗∗∗*p* < 0.001, ∗∗*p* < 0.01, ∗*p* < 0.05, ns (not significant) by One-way ANOVA. (G–I) Schematic representation of the treatment experimental design. Naive female C57BL/6 mice were inoculated s.c. with E.G7-OVA cells (5∗10^5^) in the right flank. Once tumor reached approximately 20 mm^3^, CR108, MF59, Alum plus OVA, and OVA alone were administrated near the tumor sites, respectively, with PBS as a vehicle control from days 1, 7, and 14 (G). Average tumor growth curves (H) and the survival rates were measured until day 17 (I). Data shown are mean ± SEM from two independent experiments (untreated, *n* = 9; OVA, *n* = 10; Alum + OVA, *n* = 9; MF59 + OVA, *n* = 10; CR108 + OVA, *n* = 9). ∗∗∗∗*p* < 0.0001, ∗∗∗*p* < 0.001, ∗∗*p* < 0.01, ∗*p* < 0.05, ns (not significant) by two-way ANOVA (H). (J–L)Experimental design for α-CD4 or α-CD8 blocking. naive female C57BL/six mice were inoculated s.c. with 5 × 10^5^ E.G7-OVA cells in the right flank. Once tumors reached 20 mm^3^, mice were intraperitoneally (i.p.) treated with 100 μg α-CD4 (GK1.5, BioXcell), 100 μg α-CD8 (2.43, BioXcell), or 100 μg rat IgG2b isotype control (LTF-2, BioXcell) on days 0, 5, 10, and 15. CR108 + OVA or PBS (untreated control) was administered near the tumor site on days 1, 7, and 13 (J). Average tumor growth curves (K) and survival rates (L) were determined. Data shown are mean ± SEM from two independent experiments (α-CD8 CR108 + OVA, *n* = 7; α-CD4 CR108 + OVA, *n* = 8; isotype CR108 + OVA, *n* = 7). Statistical significance: ∗∗∗∗*p* < 0.0001, ∗∗∗*p* < 0.001, ∗∗*p* < 0.01, ∗*p* < 0.05, ns (not significant) by two-way ANOVA (K).

Remarkably, the administration of OVA+CR108 resulted in a substantial reduction in tumor size after the second immunization. In contrast, mice with tumors treated with OVA+MF59, OVA+Alum, or OVA alone exhibited tumor growth comparable to the control group treated with PBS (Figure 3B). Tumor weight on day 8 was notably lower in mice treated with OVA+CR108 after the third immunization compared to those treated with other adjuvanted OVA therapies (Figure 3C). To dissect the cellular immune response against tumors, we assessed the levels of tumor-infiltrating CD8^+^ and CD4^+^ T cells post-third immunization. OVA + CR108 treatment elicited significant increased tumor-infiltrating CD8^+^ T cells compared to other groups, while CD4^+^ T cell infiltration was minimally altered (Figures 6D and 6E). An *in vivo* cytotoxic T lymphocyte (CTL) assay further revealed that OVA + CR108 enhanced lysis of OVA_257–264_-pulsed targets after two immunizations, unlike MF59 + OVA or Alum + OVA (Figure 6F). These data indicate CR108 + OVA elicit antigen-specific CD8^+^ T cell responses capable of tumor cell killing.

To mitigate potential direct toxic effects of CR108 on tumor cells, we extended the dosing interval from 4 to 7 days (Figure 6G). Figure S4 confirmed that CR108 alone, administered every 7 days, had no anti-tumor effect. Under the modified schedule, CR108+OVA significantly inhibited tumor growth, whereas other adjuvants in conjunction with OVA could not control tumors in the modified immunization schedule (Figure 6H). Additionally, all mice treated with other adjuvanted OVA formulations succumbed by day 17, whereas the survival rate of tumor models treated with OVA + CR108 was 40% (Figure 6I).

To elucidate the relative contributions of CD4^+^ and CD8^+^ T cells to the anti-tumor response, we employed α-CD4 and α-CD8 antibodies to block these cell populations during CR108 + OVA treatment (Figure 6J). Intriguingly, blocking either CD4^+^ or CD8^+^ T cells impaired the tumor control mediated by CR108 + OVA (Figures 6K and 6L). These data underscore the critical roles of both CD4^+^ and CD8^+^ T cells in the anti-tumor response elicited by CR108 + OVA treatment. Collectively, our findings demonstrate that both CD4^+^ and CD8^+^ T cells in the anti-tumor response induced by CR108 + OVA immunizations.

### CCR7-CCL19/CCL21 chemokine axis regulates Mig DCs migration and CD8^+^ T cell priming

The CCR7-CCL19/CCL21 chemokine axis is instrumental in guiding DCs from the skin to dLNs.^34^ Mig DCs, characterized by high CCR7 expression compared to Res DCs, are particularly responsive to CCL19 and CCL21 during inflammatory conditions induced by adjuvants.^35^ To investigate the impact of Mig DCs influx on CD8^+^ T cell priming, mice were pretreated via subcutaneous injection with a CCR7-blocking antibody (α-CCR7) or isotype control before immunization with OVA adjuvanted with MF59 or CR108.

**Figure 7:**
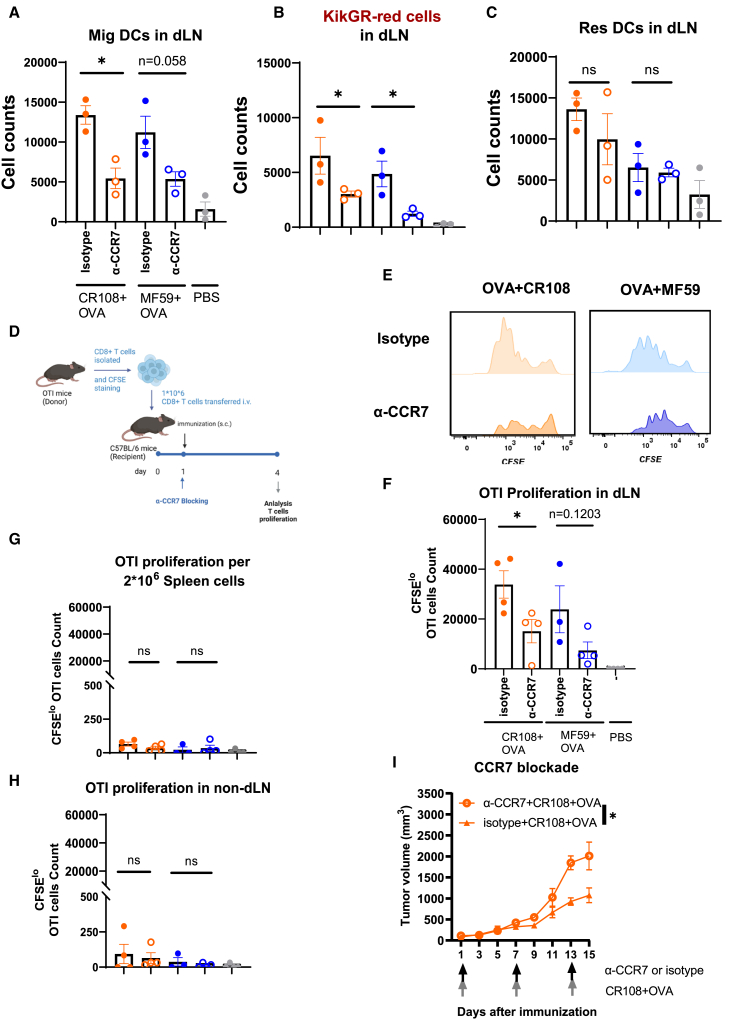
Loss of CD8^+^ T cell activation due to CCR7 blockage affecting Mig DCs recruitment (A–C) Experimental procedure: the hair-clipped skin of KikGR mice was exposed to 436 nm violet light at an intensity of 400 mW/cm^2^ for 4 min. Three hours later, 10 μg of α-CCR7 (4B12, R&D) blocking antibody per mouse was injected subcutaneously at the photoconverted skin site, while 10 μg of rat IgG2a antibody (2A3, BioXcell) per mouse was used as an isotype control. Six hours later, CR108 or MF59 plus OVA were administered subcutaneously at the α-CCR7 injection site. Forty-eight hours after administration, the numbers of (A) MHCII^hi^CD11c^med^ Mig DCs, (B) KikGR-red cells, and (C) MHCII^med^CD11c^hi^ Res DCs in the dLNs were measured by FACS. Data shown are representative of two independent experiments with three mice in each experiment (mean ± SEM). ∗*p* < 0.05, ns (not significant) by Student’s *t* test (A, B, C). (D–H) Schematic of Experimental Design: CFSE-labeled OTI CD8^+^ T cells (1 × 10^6^) were transferred into naive C57BL/six mice. One day later, the mice were subcutaneously injected with 10 μg of α-CCR7 per mouse or 10 μg of rat IgG2a antibody per mouse as an isotype control. Subsequently, the mice were immunized at the antibody-injected site with CR108 or MF59 plus OVA, and PBS as a vehicle control. Three days post-immunization, CFSE^lo^ CD45.1^+^CD3^+^CD8^+^TCRVα2^+^ T cells in dLNs, non-dLNs, and spleens were analyzed by FACS (D). Histogram analysis: histogram overlays of CFSE-dilution in CD45.1^+^ TCRVα2^+^CD8^+^ T cells from dLNs, non-dLNs, and spleens were presented. (E) Quantitative Analysis: The number of CFSE^lo^ CD45.1^+^ TCRVα2^+^CD8^+^ T cells in dLNs (F), non-dLNs (G), and spleens (H) was quantified. Data shown are representative of two independent experiments with 3–4 mice in each experiment (mean ± SEM) (isotype CR108 + OVA, *n* = 4; α-CCR7 CR108 + OVA, *n* = 4; isotype MF59 + OVA, *n* = 3; α-CCR7 MF59 + OVA, *n* = 4; PBS, *n* = 4). ∗∗∗∗*p* < 0.0001; ∗∗∗*p* < 0.001; ∗∗*p* < 0.01; ∗*p* < 0.05; ns, not significant by Student’s *t* test (F, G, H). (I) Experimental design for α-CCR7 blocking: naive female C57BL/six mice were inoculated subcutaneously with E.G7-OVA cells (1 × 10^6^) in the right flank, and tumor growth was monitored until the volume reached 20 mm^3^. To block Mig DCs migration, mice bearing E.G7-OVA tumors were treated subcutaneously with 10 μg of α-CCR7 per mouse or 10 μg of rat IgG2a antibody per mouse as an isotype control on days 1, 7, and 13. CR108 plus OVA were administered near the tumor sites, and PBS as a vehicle control (untreated), on days 1, 7, and 13. Tumor Growth Curves: Average tumor growth curves (α-CCR7 CR108 + OVA, *n* = 5; isotype CR108 + OVA, *n* = 4) were determined. Data shown are mean ± SEM from two independent experiments. ∗*p* < 0.05 by two-way ANOVA.

α-CCR7 treatment significantly reduced Mig DCs numbers in dLNs compared to isotype control (Figure 7A), while Res DCs counts remained relatively unchanged (Figure 7B). A concomitant decrease in KikGR-red cells, a marker for Mig DCs, confirmed the efficacy of α-CCR7 in inhibiting Mig DCs migration (Figure 7C).

To assess the influence of Mig DCs influx on CD8^+^ T cell priming, CFSE-labeled OT-I CD8^+^ T cells were adoptively transferred into mice followed by α-CCR7 or isotype treatment and subsequent immunization. Blocking Mig DCs migration led to a substantial reduction in proliferating OT-I CD8^+^ T cells in the dLN (Figures 7D–7F), irrespective of adjuvant type. These findings underscore the critical role of Mig DCs in CD8^+^ T cell priming within the dLN.

Consistent with the dLN data, The OT-I CD8^+^ T cells were barely observed in the spleen and non-dLNs (Figures 7G and 7H), emphasizing the primary role of dLN-derived Mig DCs in initiating CD8^+^ T cell responses.

To assess the influence of Mig DCs influx on anti-tumor efficacy, we employed α-CCR7 or isotype to block Mig DCs migration during CR108+OVA treatment. Importantly, reducing the counts of Mig DCs migration into dLN impaired the tumor control mediated by CR108+OVA (Figure 7I). These data underscore the critical roles of Mig DCs influx in the anti-tumor response elicited by CR108+OVA treatment.

These findings provide valuable insights into the intricate interplay between adjuvants and DC migration, shedding light on potential mechanisms underlying immune responses to vaccines.

## Discussion

In this study, we employed photoconvertible of KikGR mice to track skin-derived Mig DCs within dLNs following adjuvanted vaccination. Our results demonstrate a strong correlation between the quantity of KikGR-red cells and CD8^+^ T cell priming, providing a rapid and reliable method for evaluating vaccine adjuvant efficacy. CCR7 blockade with a function-blocking antibody significantly reduced KikGR-red cell influx into dLNs, resulting in impaired CD8^+^ T cell priming and diminished anti-tumor responses. These findings establish KikGR-red cells as reliable predictors of CD8^+^ T cell responses following subcutaneous immunization, particularly at 48 h. This approach offers significant advantages in terms of speed, cost-efficiency, and precision, making it highly suitable for therapeutic vaccine development.

FACS analysis identified photoconverted cells as KikGR-green^+^/KikGR-red^+^ double-positive cells (Figure S1), whereas confocal microscopy revealed red fluorescence in the photoconverted skin without detectable green fluorescence. This discrepancy arises from the duration of violet light exposure during photoconversion. As reported by Futamura et al.,^30^ prolong exposure (16 min) generates deep red fluorescence, while shorter exposure (4 min) yielded light red fluorescence. Our 4-min exposure protocol resulted in double-positive cells (KikGR-red^+^/KikGR-green^+^), but this duration was sufficient to distinguish KikGR-red (photoconverted) cells from KikGR-green (non-photoconverted) cells by FACS.

In this study, we provide significant insights into the antigen cross-presentation capabilities of Mig DCs during adjuvant-induced inflammation. Our findings demonstrate that KikGR-red cells, which serve as a proxy for Mig DCs, exhibit a significantly higher capacity to prime OT-I CD8^+^ T cells *in vitro* when activated by CR108 plus OVA, compared to those activated by MF59 plus OVA, with the latter being more effective than cells activated by Alum plus OVA. These results indicate that both the abundance and functionality of Mig DC increase in response to adjuvant stimulation, with KikGR-red cells effectively representing Mig DC activity in this context.

Furthermore, KikGR-red cells, serving as a reliable proxy for Mig DC counts, demonstrated a superior ability to prime OT-I CD8^+^ T cells *in vitro* when activated by CR108 or MF59 plus OVA, compared to Res DCs from the same immunization strategies. While recent studies have highlighted the potent capacity of Res DCs to induce T cell activation in TLR-agonist-induced inflammation, this potency was not observed in MF59- or Alum-induced inflammation.^18^^,^^36^ Our findings do not diminish the role of Res DCs in T cell immunity, but instead underscore that Mig DCs —effectively represented by KikGR-red cells— serve as a universal indicator of T cell induction across various adjuvants, including TLR agonists and other types of adjuvants during the early immune response. This highlights the critical importance of Mig DC abundance in determining an adjuvant’s effectiveness in the initial stages of immune response evaluation. Monitoring KikGR-red cell counts could therefore provide valuable insights into the immune response and the potential efficacy of adjuvants in eliciting a robust immune reaction.

CD8^+^ T cells, which function as CTLs, are critical for targeting and destroying cancer cells by inducing apoptosis through granzyme B and perforin.^37^^,^^38^ Our study found that CR108, an adjuvant, enhanced the ability of skin-derived KikGR-red cells to cross-present antigens, priming CD8^+^ T cells and generating cytotoxic effector cells crucial for tumor elimination. Interestingly, blocking CD4^+^ T cells increased tumor growth, suggesting CD4^+^ T cells support CD8^+^ T cell cytotoxicity. Recent studies highlight the role of CD4^+^ T cells in forming triads with DCs to enhance CD8^+^ T cell function during tumor eradication.^39^^,^^40^ However, the underlying mechanisms require further investigation. Notably, this does not contradict our conclusion that skin-derived KikGR-red cells predict CD8^+^ T cell responses. While regulatory T cells (Tregs) may also be involved, their roles were not assessed in this focused study. Future research will elucidate their contributions to adjuvant-enhanced immunity.

CCR7 was essential for DC trafficking to dLN during inflammation, for both Mig DCs and Res DCs.^41^^,^^42^ Our results underscore the critical role of CCR7 in DC migration to dLNs during inflammation. While both Mig and Res DCs utilize CCR7, Mig DCs exhibit enhanced CCR7 expression.^36^ Blocking CCR7 via subcutaneous injection of anti-CCR7 antibodies specifically impaired Mig DCs trafficking without affecting Res DCs migration. The consequent reduction in Mig DCs led to attenuated CD8^+^ T cell priming and compromised anti-tumor immunity. These findings emphasize the pivotal role of Mig DCs in optimizing CD8^+^ T cell responses. While CCR8 and CXCR5 contribute to DC migration, their influence on CD8^+^ T cell responses is minimal compared to CCR7 in this context. CCR8 and CXCR5 primarily facilitate the entry of allergen-activated DCs into the lymph node parenchyma, a process critical for Th2 differentiation.^43^ However, their roles do not alter the conclusion that impaired DC migration (e.g., via CCR7 deficiency) inhibits T cell priming. This highlights the dominant role of CCR7 in directing DCs to lymphoid tissues for cross-presentation, underscoring its necessity for robust CD8^+^ T cell responses.

Alum, MF59, and the TLR agonist CR108 exhibit significant differences in their mechanisms of action. Alum is known for its rapid activation of the NALP3 inflammasome,^44^ MF59 promotes antigen-specific CD8^+^ T cell responses through RIPK3-dependent signaling,^45^ and TLR agonists primarily engage MyD88 signaling to induce the production of cytokines such as IFN-I and tumor necrosis factor-α, leading to T cell immunity.^46^^,^^47^^,^^48^ The critical challenge lies in identifying a universal index that correlates with the diverse range of adjuvants.

While our study focuses on Mig DC as a predictor of CD8^+^ T cell response intensity during early innate immunity, we recognize that vaccine efficacy ultimately requires coordinated CD4^+^ T cell help, B cell maturation, and antibody production.^49^ Future studies will integrate longitudinal analyses of these adaptive immune components to establish holistic correlates of protection.

Traditional animal immunization approaches for evaluating vaccine adjuvants involve extensive experimentation, dose optimization, and lengthy efficacy studies, often requiring weeks to months to yield conclusive results.^50^^,^^51^ In contrast, our photoconvertible KikGR mouse model offers a more efficient alternative by enabling the evaluation of adjuvant-induced immune responses within just two days. This system provides a rapid and accurate assessment of the adjuvants targeting CD8^+^ T cell activation, significantly expediting the vaccine development process. The ability to rapidly identify effective adjuvants can expedite the preclinical testing phase, thereby reducing the overall time and cost associated with vaccine development. *Additionally, its adaptability to intradermal or intramuscular vaccine models expands its utility beyond subcutaneous applications, further enhancing its value in adjuvant discovery.*

The quantitative data on Mig DCs obtained through our system can be directly correlated with levels of protective efficacy. Our study demonstrated a strong positive association between the accumulation of KikGR-red cells in dLNs and the enhanced antitumor efficacy of a potent adjuvanted antigen. By monitoring the dynamics of KikGR-red cells, we can predict the effectiveness of an adjuvant in eliciting a robust CD8^+^ T cell response and subsequent protective immunity. This predictive capability is invaluable for screening and optimizing adjuvants in the early stages of vaccine development. Furthermore, the use of quantitative data enables a more precise determination of dose-response relationships, thereby improving the accuracy of dose optimization studies.

One of the key advantages of our system is its ability to quantitatively measure Mig DCs. The photoconversion technique allows for precise tracking of skin-derived Mig DCs as they migrate to the dLNs. By quantifying the number of KikGR-red cells, we could establish a robust framework for AI-driven, in-depth exploration of adjuvanted-antigen-induced CD8^+^ T cell responses. This quantitative approach provides a more objective, reproducible, and rapid evaluation of adjuvant effectiveness across different immune response pathways compared to traditional qualitative assessments.

### Limitations of the study

Although the above advantages are mentioned, several limitations of this model should be considered. First, the photoconversion system, while highly effective for tracking Mig DCs, may not be as easily adaptable to other immune cell types. For instance, photoconversion is optimized for skin-derived cells but may face challenges when applied to immune cells from deeper tissues or those that do not migrate in a similar fashion as Mig DCs. This could limit the broader applicability of the model to certain immune responses, particularly those involving tissue-resident or non-migratory cells. While the KikGR-red cells provide a robust predictor of CD8^+^ T cell activation, the long-term durability of these signals in chronic or repeated immunization contexts remains unclear. Future studies should explore how photoconversion signals behave over extended time periods and whether they can reliably reflect immune responses in such scenarios.

In summary, the photoconvertible KikGR mouse model offers several advantages over traditional animal immunization approaches. It provides a more efficient and time-saving method for evaluating vaccine adjuvants, quantitatively measures migrating DCs, enables the assessment of protective efficacy using quantitative data, and holds potential for the development of mathematical models of immune responses. These advancements have the potential to revolutionize the field of vaccine development, facilitating the rapid and accurate identification of effective adjuvants and improving the overall efficiency of the vaccine development process. By leveraging the capabilities of this system, we can accelerate the development of vaccines against emerging infectious diseases and improve the efficacy of therapeutic vaccines for chronic infections and cancer.

## Resource availability

### Lead contact

Further information and requests for resources and reagents should be directed to and will be fulfilled by the lead contact, Bin Wang (bwang3@fudan.edu.cn).

### Materials availability

This study did not generate new unique reagents.

### Data and code availability

• All source data are included in the supplementary tables.
• This paper does not report original code and sequencing data.
• Any additional information required to reanalyze the data reported in this paper is available from the lead contact upon request.

## Acknowledgments

The authors thank Mr. Jiashuo Zhang and Ms. Zhenrui Liu from the Fudan-Advaccine Joint Laboratory, as well as Ms. Xiao Guo from the Joint Live Small Animal Imaging Laboratory at Fudan University Shanghai Medical College-Revvity Company, for their excellent technical support. We also appreciate Dr. Jianhua Li from Shanghai Medical College for providing C57BL/6-Tg (TcraTcrb)1100Mjb/J (OT-I) mice and B6.SJL-Ptprca Pepcb/BoyJ (CD45.1) mice, and Dr. Minghui Zhang from Tsinghua University for supplying the E.G7-OVA cell line. The illustrations were generated using BioRender.com. This project was partly supported by a grant from the 10.13039/501100002855Ministry of Science and Technology (China) of China for the major project on Pathogenesis and Epidemic Prevention Technology System (2021YFC2302500 and 2021YFC2302501), as well as grants from the Chinese Natural Science Foundation (81991492), the Shanghai Municipal Science and Technology Major Project (ZD2021CY001), the Technician Research Projects in the School of Basic Medical Sciences at 10.13039/501100003347Fudan University (JCJSY-2024-4), and an Innovation Grant from the Fudan-Advaccine Joint Laboratory.

## Author contributions

Y.W.Z. and B.W. conceived the idea and designed the experiments. Y.W.Z., M.Y.C., H.Z.L., C.Z., and Y.H. performed the experiments. Y.W.Z. and B.W. wrote and edited the manuscript. B.W. supervised the project.

## Declaration of interests

The authors declare that some of the content from this study has been used to file a patent application. Y.W.Z., B.W., and Y.H. are listed as the inventors on China Patent no. 202411287254.6.

## STAR★Methods

### Key resources table

**Table:** 

REAGENT or RESOURCE	SOURCE	IDENTIFIER
**Antibodies**		

Rat anti-IA/IE(PerCP/cy5.5); clone M5/114.15.2	Biolegend, San Diego, CA, USA	Cat#107626; RRID:AB_2191071
Rat anti-IA/IE (BV421); clone M5/114.15.2	Biolegend	Cat#107635; RRID:AB_2561397
Armenian Hamster anti-CD11c (BV605); clone N418	Biolegend	Cat#117334; RRID:AB_2562415
Armenian hamster anti-CD11c (APC); clone N418	Biolegend	Cat#117310; RRID:AB_313779
Mouse anti-OVA257-264 bound to H-2Kb (APC); clone eBio25-D1.16	Invitrogen, San Diego, CA, USA	Cat# 17-5743-82; RRID:AB_1311286
Mouse anti-CD45.1(PE); Clone A20	Biolegend	Cat#110707; RRID:AB_313496
Armenian hamster anti-CD3 (BV510); Clone 145-2C11	Invitrogen	Cat# 11-0031-85; RRID:AB_464883
Rat anti-CD8α (BV650); Clone 53-6.7	Invitrogen	Cat# 12-0081-82; RRID:AB_465530
Rat anti-TCR Vα2(APC); Clone B20.1	Biolegend	Cat#127809; RRID:AB_1089251
Rat anti-CD45(AF700); Clone 30-F11	Invitrogen	Cat# 56-0451-82; RRID:AB_891454
Rat anti-CD3(BV421); Clone 17A2	Biolegend	Cat#100228; RRID:AB_2562553
Rat anti-CD8α (PerCP/cy5.5); Clone 53.6.7	Biolegend	Cat#100734; RRID:AB_2075238
Rat anti-mouse CCR7 IgG_2A_ antibody; Clone 4B12	R&D, Minneapolis, MN, USA	Cat#MAB3477; RRID:AB_2275533
Rat IgG_2A_ isotype control; Clone 54447	R&D	Cat#MAB006; RRID:AB_357349
InVivoMAb Rat anti-mouse CD4 IgG2b; Clone GK1.5	BioXcell, West Lafayette, IN, USA	Cat# BE0003-1, RRID:AB_1107636
InVivoMAb Rat anti-mouse CD8α IgG2b; clone L3	BioXcell	Cat# BE0061; RRID:AB_1125541
InVivoMAb Rat IgG2b isotype control, anti-keyhole limpet hemocyanin; clone LTF-2	BioXcell	Cat#BE0090; RRID:AB_1107780

**Chemicals, peptides, and recombinant proteins**		

Ovalbumins	Sigma-Aldrich, St. Louis, MO, USA	Cat#A5503
CFSE Cell Division Tracker Kit	Biolegend	Cat#423801
heat-inactivated (HI)-FBS	Biological Industries, Kibbutz Beit Kama, Israel	Cat#04-001-1ACS
1% penicillin-streptomycin	Biological Industries	Cat#03-031-1B
0.25% trypsin-EDTA	Gibco	Cat#25200056
MF59	Advaccine Biopharmaceuticals Co., Ltd. (Suzhou, China)	Cat#ADV810
Alum	Invivogen	Cat#vac-alu-250
R848	Advaccine	N/A
OVA257-264 peptide	MedChemExpress, Monmouth Junction, USA	Cat# HY-P5406
RPMI 1640 medium	Meilunbio, Shanghai, China	Cat#PWL004

**Critical commercial assays**		

MojoSort™ Mouse CD8 T cell Isolation Kit	Biolegend	Cat#480007

**Experimental models: Cell lines**		

E.G7-OVA cells	Minghui Zhang Lab, Tsinghua University, Beijing, China	N/A

**Experimental models: Organisms/strains**		

Mouse: C57BL/6J	Vital River Laboratory Animal Technology Co., Ltd. (Shanghai, China)	Stock#219
Mouse: STOCK Tg (CAG-KikGR) 33 Hadj/J	Jackson Laboratory	Stock#013753
Mouse: C57BL/6-Tg (TcrαTcrβ)1100Mjb/J (OT-I)	Jianhua Li Lab, Fudan University, Shanghai, China	N/A
Mouse: B6.SJL-Ptprca Pepcb/BoyJ (CD45.1)	Jianhua Li Lab, Fudan University, Shanghai, China	N/A

**Oligonucleotides**		

CpG1018	Advaccine	N/A

**Software and algorithms**		

FlowJo v10.1.8	FlowJo, LLC, Ashland, USA	https://www.flowjo.com/
Prism 9	GraphPad Software, LLC, Boston, USA	https://www.graphpad.com/

### Experimental model and study participant details

Female 6- to 10-week-old C57BL/6 mice were used for all experiments and purchased from Vital River Laboratory Animal Technology Co., Ltd. (Shanghai, China). STOCK Tg (CAG-KikGR) 33 Hadj/J mice (KikGR) were purchased from Jackson Laboratory. C57BL/6-Tg (TcraTcrb)1100Mjb/J (OT-I) mice and B6.SJL-Ptprca Pepcb/BoyJ (CD45.1) mice were kindly provided by Dr. Jianhua Li (Fudan University, Shanghai, China). All mice were maintained under specific pathogen-free conditions at Fudan University and treated according to the animal welfare guidelines for laboratory animals. The protocols carried out were approved by the Institutional Animal Care and Use Committee of Fudan University.

### Method details

#### Tumor cells’ preparation and challenge

E.G7-OVA cells were gifted by Dr. Minghui Zhang (Tsinghua University, Beijing, China). E.G7-OVA cells were maintained at 37°C in a 5% CO_2_ incubator, with RPMI 1640 medium (Meilunbio, MA0215, Shanghai, China) containing 10% heat-inactivated (HI)-FBS (04-001-1ACS, Biological Industries, Kibbutz Beit Kama, Israel) and 1% penicillin-streptomycin (03-031-1B, Biological Industries) 0.25% trypsin-EDTA (25200056, Gibco, Waltham, MA, USA). Naive female C57BL/6 mice were challenged with E.G7-OVA cells at 5 × 10^6^/100 μL PBS per mouse via s.c. route. When the tumor reached to approximately 20 mm^3^, E.G7-OVA bearing-mice were randomly divided into five groups for evaluation of anti-tumor efficacy of adjuvanted OVA therapies; E.G7-OVA bearing mice were randomly divided into three groups for evaluation of the influence of CD4 and CD8 T cells blocking on anti-tumor efficacy; E.G7-OVA-bearing mice were randomly divided into two groups for evaluation of the impact of CCR7 blocking on influence anti-tumor efficacy. Tumor development was monitored every two days with a caliper. $Tumor\;volume=\left(length\times {width}^{2}\right)/2$. Mice were sacrificed by CO_2_ euthanasia followed by cervical dislocation when the tumor diameter reached 20 mm^3^.

#### Photoconversion, observation, and *in vivo* animal imaging

A hair-clipped skin of female KikGR mice was exposed to violet light at 436 nm with an intensity of 200 mW/cm^2^ for 4 min to induce photoconversion. The KikGR mouse before and after photoconversion was illuminated using a hand-held lamp (LUYOR-3415RG, LUYOR, Shanghai, China) and observed through a green filter. The KikGR-red signal could be directly observed on the exposed skin region.

Female KikGR mice were randomly divided into four groups, and then were anesthetized. Abdominal skin of each group was exposed to blue light at 0, 100, 200, or 400 mW/cm^2^ for 4 min using curing equipment with a 436 nm bandpass filter. On day 1, 2, and 3 after photoconversion, KikGR-green fluorescence was acquired at EX/EM Filter 465 nm/520 nm, and KikGR-red fluorescence was acquired at EX/EM Filter 535 nm/600 nm using an *in vivo* imaging system (IVIS Spectrum CT, PerkinElmer, Waltham, USA). KikGR-red fluorescence intensities, size, and location in the skin were quantified.

#### Immunization

To treat E.G7-OVA tumor models, female C57BL/6 mice were subcutaneously (s.c.) immunized 3 times at 2-day intervals with different treatments after the E.G7-OVA tumor exceeded 20 mm^3^. To study the DCs’ migration, female KikGR mice were photoconverted on the abdominal skin, and then injected s.c. with various treatments in the center of the KikRed region. Inguinal LN (iLN) of the mice was collected after 1, 12, 24, and 48 h, and percentage and exact number of Mig DCs and Res DCs were determined. Treatments included: PBS, 10 μg OVA (Sigma-Aldrich, St. Louis, MO, USA) alone, 10 μg OVA plus 100 μg Alum (Invivogen, San Diego, CA, USA), 10 μg OVA plus 50 μL MF59 (MF59 solution was prepared by Advaccine Biopharmaceuticals Co., Ltd., Suzhou, China)), 10 μg OVA plus CR108 Adjuvant (4 mg/mL CpG1018 and 1 mg/mL R848 solutions were manufactured by Advaccine Biopharmaceuticals as previously documented [8, 40]. The volume of injected treatment was supplemented with PBS to remain at 100 μL.

#### Draining lymph nodes isolation and flow cytometry

The draining lymph nodes were dissected from the immunized mice and homogenized through a 40 μm strainer. The single-cell suspensions in dLN were stained for 15 min with a viability marker (LiveDead-efluor780, eBioscience, San Diego, CA, USA) and the following antibodies staining. For analysis of DCs’ migration, the following antibodies were used: anti-CD11c-BV605 or APC (N418, Biolegend, San Diego, CA, USA), anti-IA/IE-PerCP/cy5.5 or BV421 (M5/114.15.2, Biolegend). To analyze DCs cross-presented OVA detected by the OVA-SIINFEKL peptide on MHC I, single cells were stained with the following antibodies: anti-CD11c-BV605 (N418, Biolegend), anti-I-A/I-E-PerCP/cy5.5 (M5/114.15.2, Biolegend), anti-OVA257-264 bound to H-2Kb-APC (eBio25-D1.16, eBioscience, San Diego, CA, USA). For the analysis of CD8^+^ OT-I T cells, the following antibodies were used: anti-CD45.1-PE (A20, Biolegend), anti-CD3e-BV510 (145-2C11, Biolegend), anti-CD8a-BV650 (53-6.7, Biolegend), and anti-TCRVa2-APC (B20.1, Biolegend). Flow cytometry was performed using the LSRFortessa (BD Biosciences, Franklin Lakes, NJ, USA) and data were analyzed using FlowJo software (BD Biosciences).

#### *In vivo* CD8^+^ T cell proliferation assay

Spleens were isolated from OT-I mice and minced with the plunger end of a syringe. Splenocyte cell suspensions were filtered through 70 mm cell strainers. CD8^+^ T cells were purified using a CD8 T cell isolation kit (Biolegend) according to the manufacturer’s protocol and stained with 5 μM CellTrace CFSE (Biolegend) in PBS for 20 min in the dark. At the end of the incubation, cells were washed twice with PBS and resuspended at a concentration of 1 x 10^6^ cells/100 μL PBS. Each naive female C57BL/6 mouse was injected intravenously with 100 μL of cell suspension as a donor mouse. Twenty-four hours later (as day 1), For evaluation of CD8^+^ T cells priming by adjuvanted-OVA, the donor mice were then randomly divided into five groups. The mice were injected intradermally with OVA (10 μg/mouse) alone or in combination with Alum (Invivogen), MF59 or CR108 (Advaccine), and PBS as a vehicle control. For evaluation of the influence of CCR7 blocking on CD8^+^ T cells priming, the donor mice were then randomly divided into five groups. The mice were injected intradermally with CCR7 blocking antibody or isotype control. 6 h later, the mice were injected intradermally at the antibody-injected site with CR108 plus OVA or MF59 plus OVA, and PBS as a vehicle control. On day 4, dLNs were harvested and the LN cells suspension was stained with anti-CD3-eFluro450 (eBioscience), anti-CD8-PerCP/cy5.5 (eBioscience), anti-CD45.1-PE (Biolegend), and anti-TCRVα-APC (Biolegend) antibodies. Adoptively transferred, CFSE-labelled OT-I cells were detected in the CD3^+^CD8^+^CD45.1^+^TCRVα2^+^ gates, respectively. Results are expressed as the absolute number of CD3^+^CD8^+^CD45.1^+^TCRVα2^+^CFSE^lo^ cells (i.e., cells undergoing at least one division cycle) or percentage of each division peak within the CD3^+^CD8^+^CD45.1^+^TCRVα2^+^CFSE^lo^ gates.

#### Tumor-infiltrating T cell count

The mouse tumors were resected and digested for 45 min at 37°C in a digestion buffer containing 1000 U/mL DNase I (10104159001, Roche, Mannheim, Germany) and 2 mg/mL collagenase D (11088882001). The digested tumors were minced with a syringe plunger end to create a single-cell suspension and filtered through 70 mm cell strainers (Fisher Scientific, Waltham, MA, USA). The tumor cell suspension was stained with anti-CD45-AF700, anti-CD3-BV421, and anti-CD8-PerCP/cy5.5 for 15 min in the dark. The quantity of infiltration tumor T cells in the samples was determined by flow cytometry on the LSRFortessa (BD Biosciences).

#### Cytotoxic T lymphocyte (CTL) assay *in vivo*

Naive female C57BL/6 mice were subcutaneously immunized with CR108, MF59, Alum plus OVA, OVA alone, or PBS on days 0 and 14. On day 21 after the first immunization, the mice were sacrificed, and splenocytes were harvested. The harvested splenocytes were divided into two aliquots and pulsed with either 10 μg/1 × 10^7^ cells/mL of OVA_257–264_ peptide (MedChemExpress, Monmouth Junction, USA) or complete medium for 1 h at 37°C. The OVA_257–264_ peptide-pulsed cells were stained with 5 μM CFSE (CFSE^hi^) (Thermo Fisher Scientific), while the medium-treated cells were stained with 0.5 μM CFSE (CFSE^lo^). The two aliquots, each containing 2 × 10^7^ cells/mL, were then mixed and administered intravenously into control or immunized mice. After 20 h, the spleen was isolated from the treated mice, and splenocytes were collected and analyzed by flow cytometry. The percentages of CFSE^hi^ and CFSE^lo^ cells were determined. The percentage of OVA_257–264_-specific lysis was calculated using the following equation: $Specific\;lysis\;\left(\%\right)=\left[\left(Average\;of\;Control\;{CFSE}^{hi\;}{-Sample\;CFSE}^{hi}\right)\;/\;Average\;of\;Control\;CFSE^{hi}\right]\times 100\%$

#### Mig DCs sorting and cocultured with T cells *in vitro*

The draining lymph nodes were dissected from the immunized KikGR mice and homogenized through a 40 μm strainer (Fisher Scientific). Single cells were stained with anti-CD11c-BV605 and anti-I-A/I-E-PerCP/cy5.5 for 15 min. Flow cytometric cell sorting was performed using the BD FACSAria II (BD Biosciences). The T cells were isolated using the same method as described in the *in vivo* CD8^+^ T cell proliferation assay, including purification with the CD8^+^ T cell Isolation Kit and subsequent CFSE staining. The sorted Mig DCs and T cells were separately resuspended in RPMI 1640 medium containing 10% FBS, 1% penicillin, and 1% streptomycin and plated in 96-well plates. The co-cultured cells were then incubated at 37°C with 5% CO_2_ for 3 days, followed by staining with anti-CD11c-BV605 and anti-MHC II-PercP/cy5.5 antibodies for flow cytometric analysis.

#### *In vivo* cell depletions

To block Mig DCs accumulation into dLN, injections were administered subcutaneously (s.c.) with CCR7 function-blocking antibodies (R&D Systems, Minneapolis, MN, USA) and Rat IgG2A isotype control (R&D Systems) at a dose of 10 μg/100 μL on day 0, and then detection of the number of KikGR-red cells, Mig DCs, and Res DCs on day 2. To determine the CD8^+^ T cells priming after α-CCR7 injection, The CFSE-labeled CD8^+^ OT-I T cells were injected intravenously into C57 mice at a concentration of 1 x 10^6^cells/100 μL at day −2 and injected s.c. with CCR7 function-blocking antibodies (R&D, Systems) and Rat IgG2A isotype control (R&D Systems) at a dose of 10 μg/100 μL at day-1. These C57 mice were divided into several groups and subsequently immunized with PBS, 10 μg OVA plus 50 μL MF59, and 10 μg OVA plus CR108 (20 μg each of CpG1018 and R848) on day 0, respectively. After 72 h of the immunization, single-cell suspensions of draining lymph nodes and spleens were harvested and stained with anti-CD3-eFluor450, anti-CD8-PercP/cy5.5, anti-CD45.1-PE, and anti-TCRVα-APC for flow cytometric analysis. To CD4^+^ or CD8^+^ T cell depletion, the mice bearing E.G7-OVA tumors were treated with 100 μg of αCD4 (GK1.5, BioXcell, West Lafayette, IN, USA) i.p. on days 0, 5, 10, and 14 or 100 μg of αCD8 (2.43, BioXcell) i.p. on days 0, 5, 10, and 14. CR108, MF59, Alum plus OVA, and OVA alone were administered near the tumor sites, respectively, with PBS as a vehicle control on days 1, 7, and 14. Average growth curves and animal survival were determined.

### Quantification and statistical analysis

Data are presented as mean ± SEM. Statistical analysis was conducted using GraphPad Prism software. Differences in mean values between groups were assessed using the Student’s t test, one-way ANOVA or two-way ANOVA as indicated in the figure legend. Significance levels are as follows: ∗∗∗∗*p* < 0.0001; ∗∗∗*p* < 0.001; ∗∗*p* < 0.01; ∗*p* < 0.05; not significant, *p* > 0.05. Data points in all figures represent independent lymph nodes, unless otherwise specified.
