# Supplementary material for: Photoconverted cells allow rapid assessment of vaccine adjuvant potency in mice
Source: iScience. 2025 Jun 5;28(7):112774. doi: 10.1016/j.isci.2025.112774 (PMC12221512; doi:10.1016/j.isci.2025.112774)
Supplement: Document S1. Figures S1–S4 [file mmc1.pdf]

**iScience, Volume *28***

## Supplemental information

# Photoconverted cells allow rapid assessment of vaccine adjuvant potency in mice

**Yiwei Zhong, Mingyue Chen, Hongzhe Lin, Cheng Zu, Yue He, and Bin Wang**

# Supplementary Figures

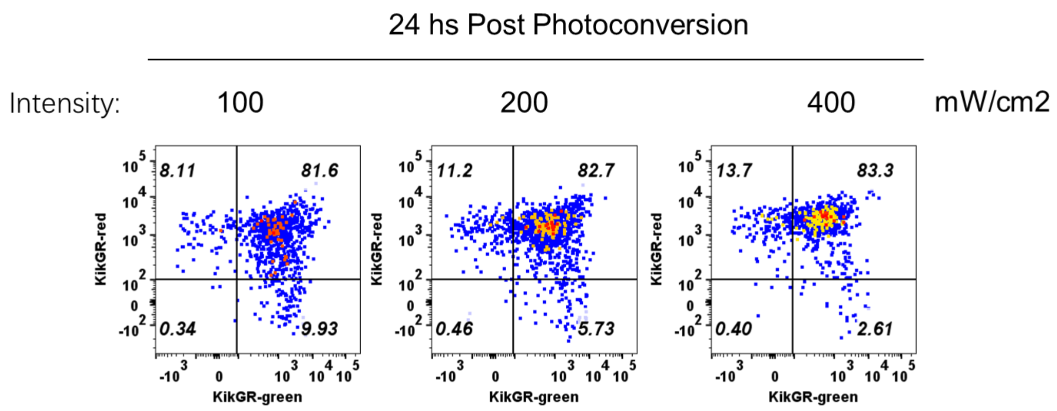


**Supplementary Figure 1. Detection of CD45+ cells in photoconverted skin following elevated light intensity.**

The hair-clipped skin of KikGR mice were photoconverted by violet light at intensities of 100, 200, and 400 mW/cm$^2$, respectively. The photoconverted skin region were collected, processed into single-cell suspensions, and analyzed for CD45+ cells with KikGR-red or KikGR-green fluorescence using flow cytometry.

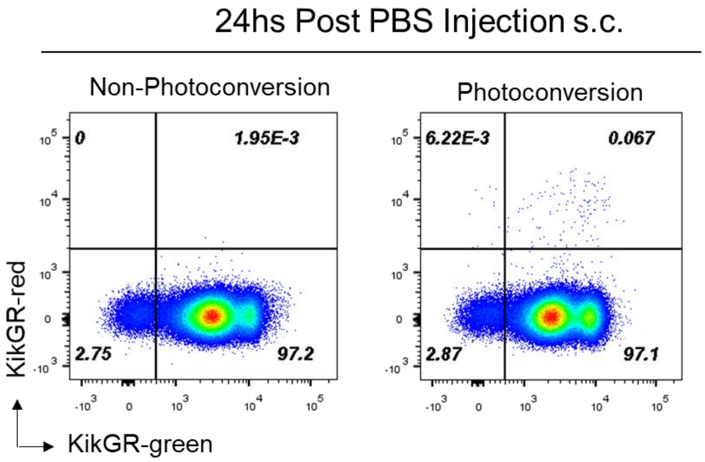


**Supplementary Figure 2. Detection of unconverted negative controls post PBS injection.**

The hair-clipped skin of KikGR mice were randomized divided in two groups. The one were converted by violet light at intensities of 400 mW/cm$^2$ , and the other were unconverted. Three hours later, all mice were subcutaneously injected with PBS. After 24 hours, draining lymph nodes (dLNs) were harvested and analyzed for the presence of KikGR-red+ cells using FACS.

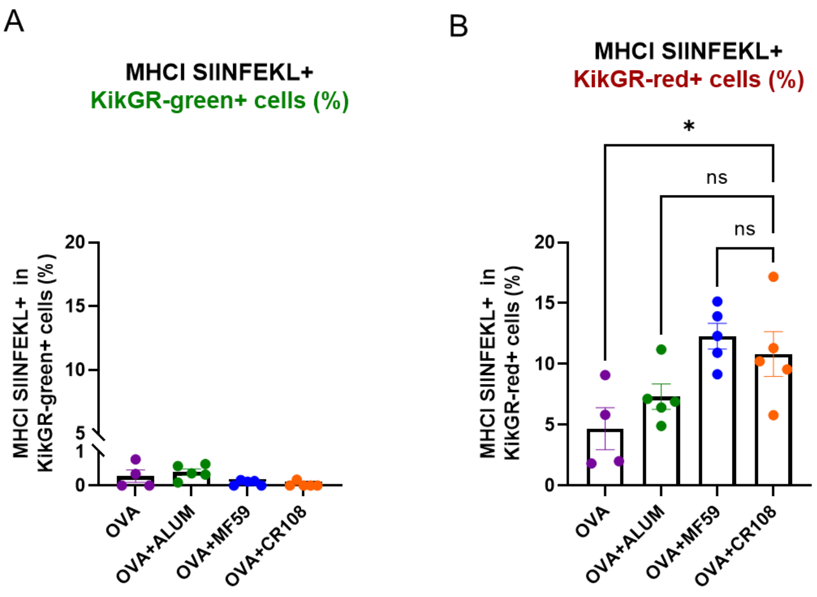


**Supplementary Figure 3 KikGR-green cells exhibit a lower cross-antigen presentation capacity compared to KikGR-red cells，related to Figure 5.**

The hair-clipped skin of KikGR mice was exposed to 436 nm violet light at an intensity of 400 mW/cm² for 4 minutes. Three hours post-exposure, the photoconverted skin was treated with CR108+OVA, MF59+OVA, Alum+OVA, OVA alone, or PBS as a vehicle control. The expression level of the MHC-I OVA257-264 (SIINFEKL) complex in various adjuvants plus OVA（excluding PBS) was evaluated on total KikGR-green+ cells **(A)** and total KikGR-red+ cells **(B)** were analyzed within dLNs 48 hours after treatment using flow cytometry. Data shown are representative of two independent experiments with 4-5 mice in each experiment (mean ± SEM) (OVA, n=4; OVA+Alum, n=5; OVA+MF59, n=5; OVA+CR108, n=5). *$p < 0.05$; n, not significant by One-way ANOVA (A, B).

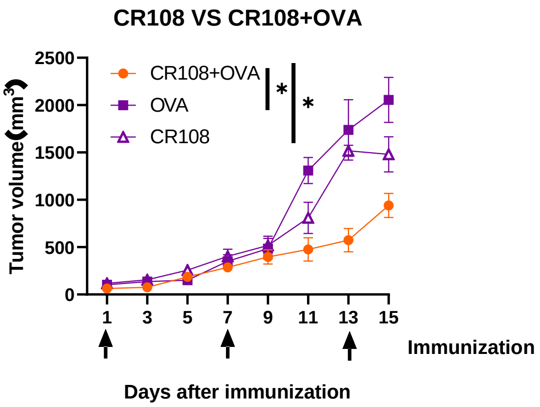


**Supplementary Figure 4. The anti-tumor efficacy not cause by the adjuvant's direct toxicity to tumor cells.**

Naïve female C57BL/6 mice were inoculated s.c. with E.G7-OVA cells (5*10^5) in the right flank. Until the tumor reached to 20 $mm^3$, CR108 plus OVA (n=4), CR108 alone (n=4), and OVA alone (n=5) were administrated near the tumor sites, respectively, from days 1, 7, and 14. Average growth curves show mean ± SEM.
